# Robust Hydrophobic and Hydrophilic Polymer Fibers Sensitized by Inorganic and Hybrid Lead Halide Perovskite Nanocrystal Emitters

**DOI:** 10.3389/fchem.2019.00087

**Published:** 2019-02-26

**Authors:** Paris G. Papagiorgis, Andreas Manoli, Androniki Alexiou, Petroula Karacosta, Xenofon Karagiorgis, Georgia Papaparaskeva, Caterina Bernasconi, Maryna I. Bodnarchuk, Maksym V. Kovalenko, Theodora Krasia-Christoforou, Grigorios Itskos

**Affiliations:** ^1^Experimental Condensed Matter Physics Laboratory, Department of Physics, University of Cyprus, Nicosia, Cyprus; ^2^Department of Mechanical and Manufacturing Engineering, University of Cyprus, Nicosia, Cyprus; ^3^Empa - Swiss Federal Laboratories for Materials Science and Technology, Dübendorf, Switzerland; ^4^Laboratory for Inorganic Chemistry, Department of Chemistry and Applied Biosciences, ETH Zürich, Zurich, Switzerland

**Keywords:** perovskites, lead halides, nanocrystals, electrospinning, polymer fibers

## Abstract

Advances in the technology and processing of flexible optical materials have paved the way toward the integration of semiconductor emitters and polymers into functional light emitting fabrics. Lead halide perovskite nanocrystals appear as highly suitable optical sensitizers for such polymer fiber emitters due to their ease of fabrication, versatile solution-processing and highly efficient, tunable, and narrow emission across the visible spectrum. A beneficial byproduct of the nanocrystal incorporation into the polymer matrix is that it provides a facile and low-cost method to chemically and structurally stabilize the perovskite nanocrystals under ambient conditions. Herein, we demonstrate two types of robust fiber composites based on electrospun hydrophobic poly(methyl methacrylate) (PMMA) or hydrophilic polyvinylpyrrolidone (PVP) fibrous membranes sensitized by green-emitting all-inorganic CsPbBr_3_ or hybrid organic-inorganic FAPbBr_3_ nanocrystals. We perform a systematic investigation on the influence of the nanocrystal-polymer relative content on the structural and optical properties of the fiber nanocomposites and we find that within a wide content range, the nanocrystals retain their narrow and high quantum yield emission upon incorporation into the polymer fibers. Quenching of the radiative recombination at the higher/lower bound of the nanocrystal:polymer mass ratio probed is discussed in terms of nanocrystal clustering/ligand desorption due to dilution effects, respectively. The nanocomposite's optical stability over an extended exposure in air and upon immersion in water is also discussed. The studies confirm the demonstration of robust and bright polymer-fiber emitters with promising applications in backlighting for LCD displays and textile-based light emitting devices.

## Introduction

Lead halide perovskite nanocrystals (LHP NCs) of the APbX_3_-type with A being cesium (Cs) or formamidinium (FA) and X being a halide ion (Cl, Br, I) have emerged as outstanding light emitting materials (Protesescu et al., 2015, 2016, 2017; Kovalenko et al., 2017; Akkerman et al., 2018), exhibiting tunable, bright emission across the visible spectrum with excellent optical amplification properties, confirming their high potential for photonic applications (Yakunin et al., 2015; Sutherland and Sargent, 2016). Realization of practical LHP NC emitters is critically dependent upon improving their structural, thermodynamical, and optical stability under ambient conditions, which remains the foremost challenge for such nanomaterials (Huang et al., 2017; Kovalenko et al., 2017; Akkerman et al., 2018; Zhao et al., 2018). Encapsulation of perovskite NCs into macro- or nanoscale polymeric structures has been recently demonstrated (Huang et al., 2016; Raja et al., 2016; Wang et al., 2016, 2017; Hou et al., 2017; Lu et al., 2017; Ma et al., 2017; Murphy et al., 2017; Demkiv et al., 2018; Liao et al., 2018; Lin et al., 2018; Sygletou et al., 2018; Tsai et al., 2018; Wong et al., 2018; Xin et al., 2018; Yang M. et al., 2018; Yang S. et al., 2018; Zhang et al., 2018; Zhu et al., 2018) as simple and low-cost methodologies to preserve the LHP NC chemical integrity by suppressing water and oxygen transmission, improving the thermal stability, and reducing structural modifications, to which LHP NCs are highly susceptible, such as ligand desorption and nanocrystal sintering. Furthermore, integration of the NCs into polymers provides a method to improve their solid-state processability into films, microspheres, fibers, or more complex composites while offering new functionalities such as polarizing PL (Raja et al., 2016; Lu et al., 2017), light detection (Wang et al., 2016), biological labeling and sensing (Wang et al., 2017; Zhu et al., 2018) or device applications such as light emitting diodes (Huang et al., 2016; Liao et al., 2018; Lin et al., 2018; Tsai et al., 2018; Xin et al., 2018; Yang M. et al., 2018; Zhang et al., 2018; Zhu et al., 2018).

All the studies above employ Cs-based all-inorganic NC sensitizers with the notable exception of refs (Lu et al., 2017; Liao et al., 2018; Yang S. et al., 2018), in which methylammonium lead halide NCs were utilized. On the other hand, the polymer encapsulation of the closely related hybrid analog, but more chemically robust and equally bright formamidinium lead halide NCs (Protesescu et al., 2016, 2017) has not received much attention. In addition, a particularly important parameter for such polymer-nanocrystal emitters is the influence of the relative polymer-nanocrystal concentration on the optical properties of the composite which has not been thoroughly investigated. Motivated by these considerations, we present herein a thorough investigation of electrospun polymer fiber nanocomposites sensitized with green-emitting all-inorganic CsPbBr_3_ and hybrid organic-inorganic FAPbBr_3_ NCs. Electrospinning allows for a simple, cost-effective and industrially scalable fabrication of long and continuous polymer fibers (Persano et al., 2013; Yu et al., 2017; Savva and Krasia-Christoforou, 2018), enabling the incorporation of a variety of inorganic nanoparticles and thus generating functional nanocomposites (Chronakis, 2015). Fiber composite emitters appear particularly attractive for optoelectronics bearing unique properties such as tolerance to extremely small bending radii and large tensile strains while preserving the structural integrity and bright emission of the perovskite NCs (Wang et al., 2016, 2017; Murphy et al., 2017; Liao et al., 2018; Lin et al., 2018; Tsai et al., 2018; Yang M. et al., 2018). In particular, enhanced water/optical (Wang et al., 2016, 2017; Murphy et al., 2017; Liao et al., 2018; Lin et al., 2018; Tsai et al., 2018; Yang M. et al., 2018) and thermal (Liao et al., 2018) stabilities have been demonstrated upon nanocrystal encapsulation into polymer fibers. Elaborate core/shell NC-fiber geometries with new functionalities have been produced (Murphy et al., 2017; Yang M. et al., 2018;) while applications such as white light emitting diodes (Liao et al., 2018; Lin et al., 2018; Tsai et al., 2018; Yang M. et al., 2018;), fluorescence detection (Wang et al., 2016), and biological/metal ion sensing (Wang et al., 2017) have also been reported. We demonstrate herein the realization of two robust NC-polymer fiber emitter types via the following: (i) encapsulation of the perovskite NCs into hydrophobic poly(methyl methacrylate) (PMMA) fibers using an electrospinning process, and (ii) NC coating hydrophilic cross-linked polyvinylpyrrolidone (PVP) fibrous membranes via a simple immersion process. A thorough investigation of the structure-property relations with emphasis on the optical properties upon varying the NC concentration over three orders of magnitude is reported and compared with the respective characteristics of reference pristine and polymer-NC blend films. Air- and water-stability luminescence experiments confirm the attractive encapsulating properties of PMMA fibers, confirming the demonstration of robust, bright and versatile polymer-fiber emitters.

## Materials and Methods

### Synthesis of CsPbBr_3_ Nanocrystals

In a 25 ml three-necked flask, PbBr_2_ (69 mg, 0.188 mmol, ABCR) was suspended in octadecene (5 ml, Sigma-Aldrich), dried at 100°C for 30 min and mixed with oleic acid (0.5 ml, Sigma-Aldrich, vacuum-dried at 100°C) and oleylamine (0.5 ml, STREM, vacuum-dried at 100°C). When PbBr_2_ was dissolved, the reaction mixture was heated up to 180°C and preheated cesium oleate in octadecene (0.4 ml, 0.125 M) was injected. The reaction mixture was cooled immediately with an ice bath to room temperature.

#### Purification and Size-Selection of CsPbBr_3_ Nanocrystals

The crude solution was centrifuged at 12,100 rpm for 5 min, following which the supernatant was discarded, and the precipitate was dissolved in 300 μl hexane. The hexane solution was centrifuged again and the precipitate was discarded. The supernatant was diluted two times and used for the subsequent DDAB-treatment.

#### DDAB-Treatment of CsPbBr_3_ Nanocrystals

0.6 milliliter toluene was added to CsPbBr_3_ NC colloidal solution prepared as described above. Then 30 μl of oleic acid and 160 μl of DDAB (didodecyldimethylammonium bromide, 0.05 M in toluene) were added to the colloidal solution of CsPbBr_3_ NCs. The mixture was stirred for 1 h, followed by the precipitation with 1.8 ml of ethyl acetate and centrifuged at 12.1 krpm for 3 min. The precipitate was redispersed in 0.5 ml toluene and this solution was additionally filtered through a 0.45-μm PTFE-filter.

### Synthesis of FAPbBr_3_ Nanocrystals

#### Preparation of Oleylammonium Bromide (OAmBr)

Oleylamine (12.5 ml, Acros Organics, 80–90%) and ethanol (100 ml, Aldrich) were mixed in a 250 ml flask. The reaction mixture was cooled in an ice-water bath and 8.56 ml HBr (48% aqueous solution, Aldrich) was added. The reaction mixture was left to react overnight under nitrogen flow. Then the solution was dried in a rotary evaporator and the obtained product was washed multiple times with diethyl ether and then a white powder was dried under vacuum at room temperature for several hours. OAmBr was stored in the glovebox.

#### Synthesis of FAPbBr_3_ Nanocrystals

In a 25 ml three-necked flask, lead (II) acetate trihydrate (76 mg, 0.2 mmol, Sigma-Aldrich) and formamidinium acetate (78 mg, 0.75 mmol, Sigma) were suspended in octadecene (8 ml) and oleic acid (2 ml, Sigma-Aldrich), heated to 50°C and then dried under vacuum for 30 min. Then the reaction mixture was heated to 130°C and, at this point, the mixture of 266 mg (0.8 mmol) of OAmBr in anhydrous toluene (2 ml) was injected into the reaction flask (to dissolve OAmBr in toluene, a mixture of them should be preheated at 40–50°C). After another 1 min, the reaction mixture was cooled by an ice-water bath.

#### Isolation and Purification of Nanocrystals

Sixteen milliliter of methyl acetate (ABCR) were added to the crude solution followed by the centrifugation at 12.1 krpm for 5 min (Centrifuge: Eppendorf 5804) and the supernatant was discarded. The precipitate was dissolved in toluene (5 ml) and the solution was centrifuged again (3 krpm, 2 min). The supernatant, containing monodisperse nanocrystals, was retained for the DDAB-treatment, while the precipitated NCs were discarded.

#### DDAB-Treatment of FAPbBr_3_ Nanocrystals

Five milliliter toluene was added to FAPbBr3 colloidal solution prepared as described above. Then 0.1 ml of oleic acid and 0.6 ml of DDAB (didodecyldimethylammonium bromide, 0.05 M in toluene) was added to 10 ml colloidal solution of FAPbBr3 nanocrystals. The mixture was stirred for 1 h, followed by the precipitation with 16 ml of ethyl acetate and centrifuged at 12.1 krpm for 3 min. Precipitate was re-dispersed in 5 ml toluene and this solution was additionally filtered through a 0.45-μm PTFE-filter.

### PMMA/LHP Fibers

Poly(methyl methacrylate) (PMMA, Mn = 350,000 g/mol) was obtained from Sigma-Aldrich and used without further purification. Chloroform (CHCl_3_, reagent grade, Scharlau) was the solvent employed in the preparation of the PMMA/LHP electrospun fiber emitters. Initially, a homogeneous solution of PMMA was prepared in CHCl_3_ (solution concentration 20% w/v). Subsequently, 2 mL of the above solution was mixed together with 134 μL of the CsPbBr_3_ and FAPbBr_3_ perovskite nanocrystal solutions prepared in toluene, as described in sections Synthesis of CsPbBr_3_ Nanocrystals and Synthesis of FAPbBr_3_ nanocrystals (0.33% wt in respect to the polymer mass). The same procedure was repeated to obtain a series of PMMA/LHP (FAPbBr_3_) solutions having different nanocrystal content, i.e., 0.05% wt (~20 μL), 0.1% wt (~40 μL), 0.22% wt (~80 μL), 0.66% wt (~242 μL), 1% wt (~367 μL), and 5% wt (~2 ml). The obtained solutions were then loaded into a 10 mL glass syringe in the electrospinning set-up to be electrospun. All electrospinning experiments were performed at room temperature. Equipment included a controlled-flow, four-channel volumetric microdialysis pump (KD Scientific, Model: 789252), syringes (16G) with specially connected spinneret needle electrodes, a high-voltage power generator (10–50 kV, ES50P-20W Gamma High Voltage Research) and a custom-designed grounded target collector (282 mm length × 279 mm height), inside an interlocked Faraday enclosure safety cabinet. The electrospinning conditions employed in all cases were the following: Flow rate: 1.4 ml/h; applied voltage: 17 kV; needle-to-collector distance: 12 cm.

### PVP/LHP Fibers

Polyvinylpyrrolidone (PVP, Mn = 1,300,000 g/mol) was obtained from Sigma-Aldrich and used without further purification. A PVP polymer solution prepared in methanol (CH_3_OH, analytical grade, Scharlau) with a concentration of 25% w/v was used to electrospin the PVP fibers using the same setup as described in 2.4. The optimum electrospinning conditions to obtain uniform bead-free fibers were the following: Applied voltage: 22–33 kV; flow rate: 4.6 mL/h; needle diameter: 16G; needle-to-collector-distance: 25 cm. Subsequently, the as-prepared PVP fibers were placed in an oven and they were thermally crosslinked by following a three-step controlled heating procedure (de Mello et al., 1997; Chronakis, 2015): 1st step 100°C (3 h), 2nd step:140°C (3 h), and 3rd step: 170°C (3 h). After their fabrication, the hydrophilic PVP cross-linked fibers were functionalized with octyltriethoxysilane (96%) to increase their wettability to the hydrophobic LHP NC solution. NC loading of the functionalized fibrous membranes was achieved via their simple immersion into NC toluene solutions or via spin coating of the NC solutions onto the membrane, with the latter method yielding more uniform dispersion of the NCs within the fibers. The NC concentration within the fibers was measured by weighting before each step, namely (a) before functionalization, (b) after functionalization, and (c) after the NC deposition. Each step was executed allowing sufficient time within to ensure solvent evaporation.

### Structural Characterization

Transmission electron microscopy (TEM/STEM) images of nanocrystals were captured using a JEOL JEM-2200FS microscope operated at 200 kV. Powder X-ray diffraction (XRD) was recorded using a powder diffractometer (STOE STADI P) with Cu Kα1 radiation operated in transmission mode combined with a germanium monochromator and a silicon strip detector (Dectris Mythen). The morphological characteristics of the electrospun fibers were obtained via scanning electron microscopy (SEM) (Vega TS5136LS-Tescan). The samples were gold-sputtered (~30 nm) (sputtering system K575X Turbo Sputter Coater–Emitech) prior to SEM inspection. Fluorescence microscopy was used to probe the spatial distribution of NCs within the electrospun fibers. The samples were placed on glass slides, covered with coverslips and measured under an Olympus fluorescence microscope (U-RLF-T model). The fluorescence intensity of each sample was determined using the CY3 filter (Excitation: 450 nm, Emission: 513–556 nm). Images were taken at 20× and 100× magnifications and analyzed using the ImageJ software.

### Optical Characterization

NC film/solution absorption was carried out by a Perkin Elmer Lamda 1050 spectrophotometer equipped with a three-detector module covering the 300–3,000 nm spectral range. Steady state photoluminescence (PLE, PL) experiments were performed on a 0.35 m FluoroLog FL3 Horiba Jobin Yvon spectrofluorimeter equipped with a TBX-04 visible PMT with detection range from 250 to 850 nm. Excitation of the samples for the PLE experiments was achieved via monochromator-filtered output of a 450 W ozone-free Xe lamp. PL quantum yield (QY) experiments were executed according to reference (de Mello et al., 1997) using a 4-inch Labsphere integrating sphere, from which the light was collected via a fiber bundle and detected by a 0.75 m Acton750i Princeton spectrometer equipped with a 1024 × 256-pixel PIXIS charge-coupled device (CCD) camera with spectral response in the range of 250–950 nm. The samples were excited using an Oxxius, 375 nm, 5 mW laser diode.

Time-resolved photoluminescence (TRPL) was acquired using a monochromator-based time-correlated single-photon counting (TCSPC) method on the same setup as that used for the steady-state PL experiments. PL was excited by a laser diode at 375 nm with a pulse width of ~150 ps, operating at a repetition rate of 100–250 KHz. The beam diameter of the exciting beam was ~0.5 mm. To allow spatial averaging over the probed samples, multiple spots were probed. The PL decays were obtained while monitoring the PL emission peak with a spectral bandwidth of 10 nm. The average transient PL lifetime τ_avg_ for TRPL decays was calculated from the relation:

$$\tau_{av}=\frac{\sum_{i}A_{i}\tau_{\iota }^{2}}{\sum_{i}A_{i}\tau_{i}}$$

where τ_*i*_ are the decay times and *A*_*i*_ the respective decay amplitudes extracted from multi-exponential fits of the PL transients. All optical data were acquired in free space and ambient conditions and were corrected to take into account the spectral response of the grating and detector used.

## Results and Discussion

### PMMA-LHP NC Fibers

**Figure 1:**
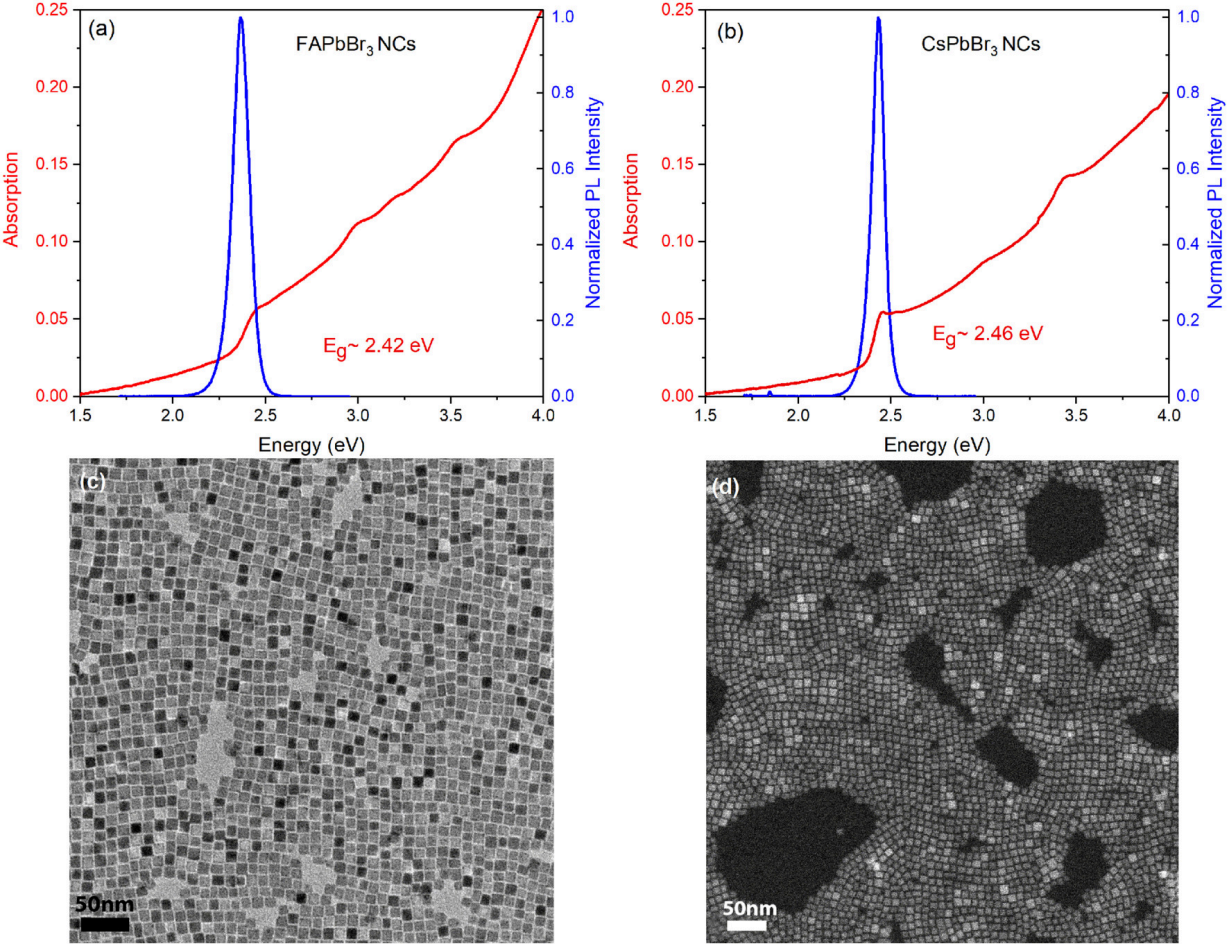
Absorption and PL spectra of the film of pristine **(a)** FAPbBr_3_ **(b)** CsPbBr_3_ NC. High resolution TEM images illustrating the cubic-shaped **(c)** FAPbBr_3_, and **(d)** CsPbBr_3_ NCs employed in our studies.

Figures 1a,b displays typical absorption and photoluminescence (PL) spectra from pristine FAPbBr_3_ and CsPbBr_3_ NC films employed in this work. Both types of nanocrystals are treated by didodecyldimethylammonium bromide ligands that have been shown to efficiently heal surface traps improving overall the robustness and the emission properties of the material (Bodnarchuk et al., 2019). The produced NCs are cubic-shaped with average sizes in the ~10–12 nm range, as seen in the high resolution TEM images of Figure 1. Powder X-ray diffraction data, presented in Figure S1, indicate that the DDAB-capped NCs exhibit an orthorhombic crystal structure. The high quality of the NC material is witnessed by the narrow linewidth, i.e., FWHM <100 meV, and the bright emission reaching quantum yields as high as 86% in the solid state.

**Figure 2:**
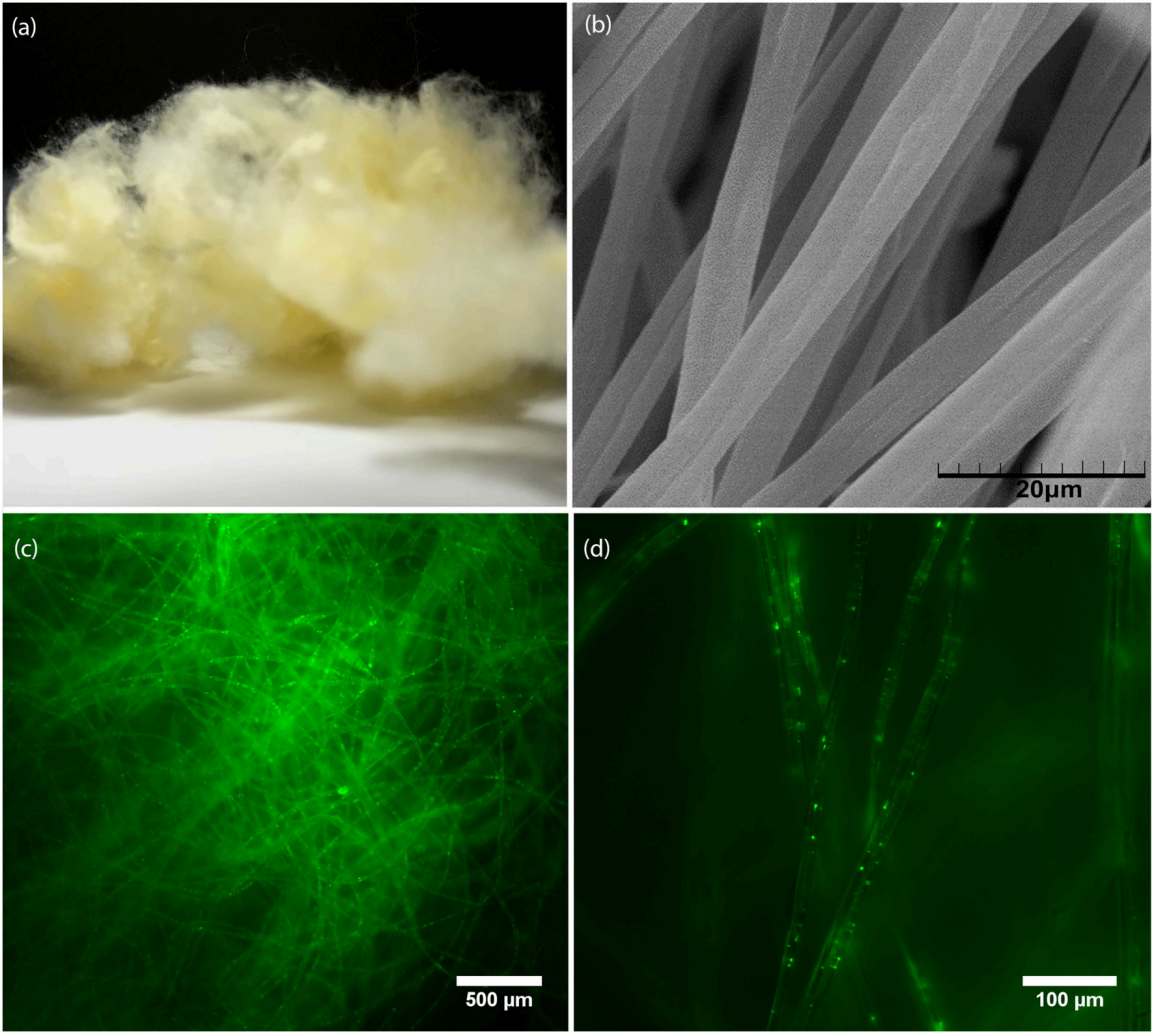
**(a)** Photograph, **(b)** SEM image, **(c,d)** Fluorescence microscopy images at different magnifications for electrospun FAPbB_r3_ NC:PMMA fibers with polymer:NC mass ratio of 100:1 (1%wt).

The macroscopic cotton-like and the microscopic fibrous morphology of an electrospun PMMA/FAPbBr_3_ NC membrane with an NC:polymer mass ratio of 1:100 are displayed in Figures 2a,b, respectively. From software analysis of the SEM images, the average diameters of fibers are estimated and found equal to ~3.3 ± 0.3 μm and ~5.5 ± 1.3 μm for fibers containing 0.1% wt FAPbBr_3_ and CsPbBr_3_ NCs, respectively. As more NCs are loaded into fibers, the average fiber diameter increases, i.e., an increase in the NC content by one order of magnitude results in an increase of the fiber diameter by ~45%, as observed in Figure S2. Figures 2c,d contain fluorescence microscopy images of the FAPbBr_3_ NC-sensitized sample at different magnifications illustrating the uniform encapsulation of NCs within the PMMA fibers. A general observation based on the spatial distribution of the emission observed, vividly illustrated in representative fluorescence images in Figure S3, is that the FA-based NCs appear to spatially disperse more orderly compared to the Cs-based NCs that tend to cluster within the PMMA fibers.

**Figure 3:**
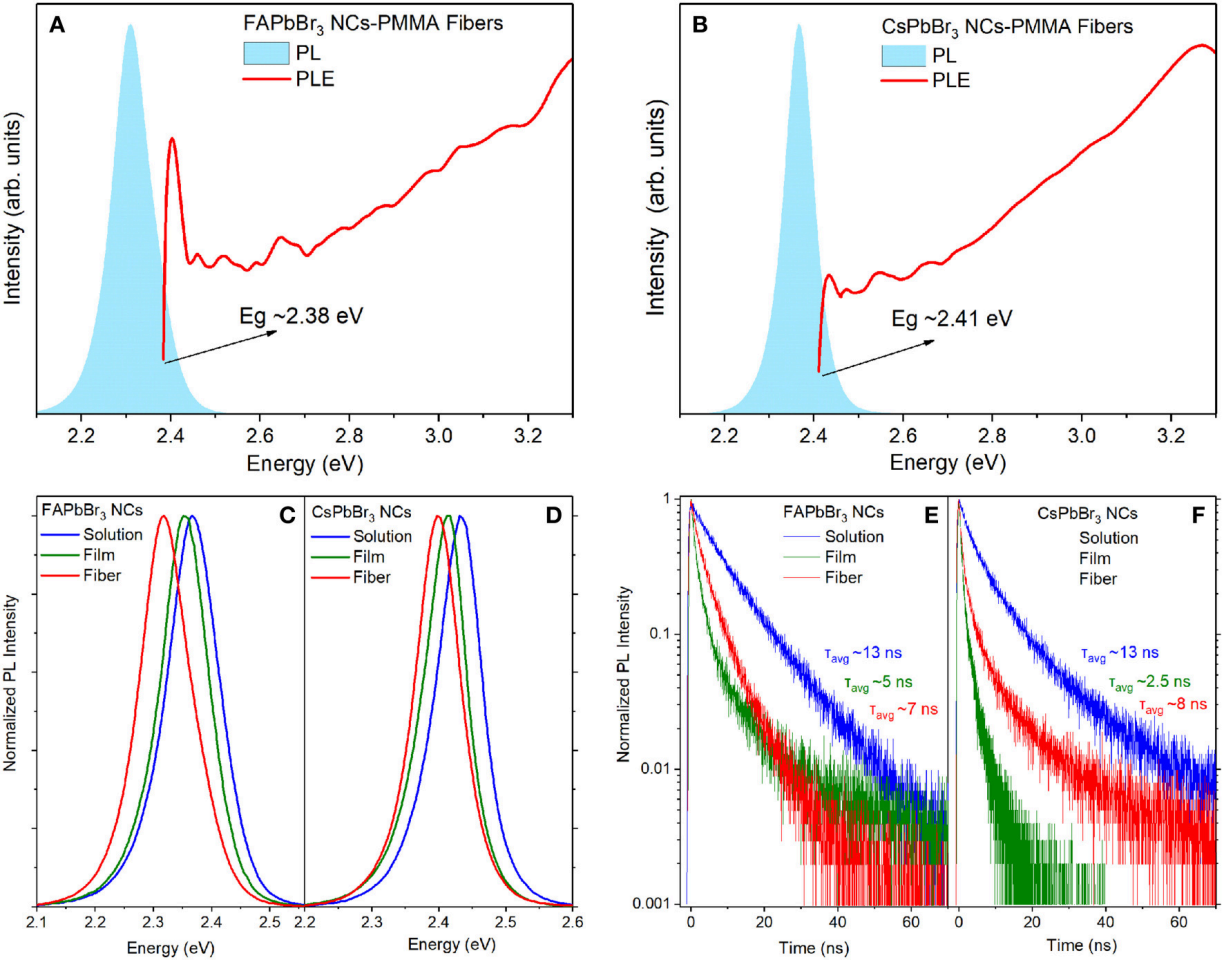
PL and PLE spectra from 0.33% wt/wt NC:PMMA fibers containing **(A)** FAPbBr_3_ NCs, **(B)** CsPbBr_3_ NCs. Comparative study of normalized PL spectra in the colloidal, solid and fibrous state for **(C)** FAPbBr_3_ NCs, **(D)** CsPbBr_3_ NCs. Transient PL decays in the three states from **(E)** FAPbBr_3_ NCs, **(F)** CsPbBr_3_ NCs.

Typical optical characteristics of the PMMA-NC membranes are displayed in Figures 3A,B for fibers containing FAPbBr_3_ and CsPbBr_3_ NCs, respectively. The membranes are opaque to visible light, so optical absorption experiments could not be employed to measure the energy gap of the fiber-embedded perovskite NCs. Instead, the optical gap of the perovskite NCs within the fibers is estimated by the onset of the band-edge luminescence identified by excitation photoluminescence (PLE) experiments, as seen in Figure 3.

In Figures 3C–F, comparative steady-state and transient PL spectra of the two NC systems are displayed in the colloidal, solid and fibrous states. PL transients in NC solutions are well-approximated with double exponentials while NC decays in the solid state, i.e., films and fibers exhibit more complex decays requiring curve fits by triple exponentials. Typically, the decays are dominated by a short decay of ~2–4 ns that is assigned to the radiative recombination while weaker and longer component(s) in the ~8–40 ns range are attributed to non-radiative quenching channels via defects. The assignment is based on the observation that the relative weight of the longer PL transient appears inversely proportional to the emission quantum yield of the samples, i.e., the most efficient emitters exhibit faster PL transients with a weaker amplitude of the long-lived recombination channel. An overall characterization of the dynamics is quantified by the average PL lifetimes, computed as described in Materials and Methods section. A consistent red shift of the band-edge PL NCs, compared to the PL peak in dilute NC solutions and films, is observed upon encapsulation of both types of nanocrystals into the polymer fibers. The bathochromic (toward red) PL shift is accompanied by a slightly broader emission linewidth and average PL lifetimes decreased/increased compared to the colloid/solid samples, respectively. The progressively stronger dielectric environment as the NCs are dispersed from toluene solutions to PMMA fibers and finally to close-packed NC films can explain the trend in the observed PL dynamics; however, dielectric considerations are not consistent with the respective variation of the PL peaks in the steady-state regime.

**Figure 4:**
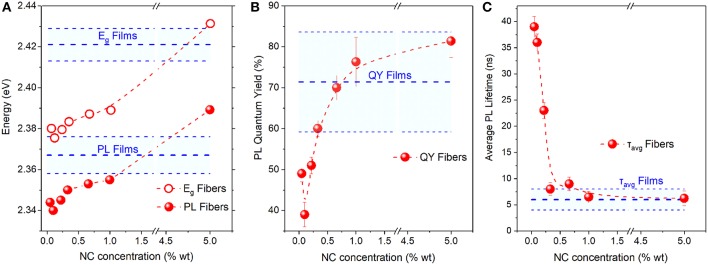
**(A)** Energy gap (E_g_) and PL Peak, **(B)** PL quantum yield, and **(C)** PL lifetime vs. NC concentration for the PMMA/FAPbBr_3_ NC composite. Respective characteristics for reference pristine NC films are also displayed; the main blue dotted line defines the mean values and the error bands/cyan highlighted area denotes the standard deviation of the PL characteristics yielded by statistics on different fibers/films. All displayed data points have been produced out of the same parent FAPbBr_3_ NC solution.

To provide further insight into the spectral and dynamical modifications of the NC emission upon incorporation into the PMMA fibers, we performed a systematic NC concentration-dependent study of their luminescence properties while varying the NC loading by two orders of magnitude and employing appropriate statistics by probing multiple fiber and reference pristine NC samples produced out of the same parent NC solution. FAPbBr_3_ NC-based membranes were selected for the study due to the better spatial dispersion of the NCs they exhibit, as discussed previously. The study summary that includes the variation of the band-edge, PL peak, PL lifetime, and PL QY with NC content is shown in Figure 4, with the relevant optical data listed in Table S1. The respective raw PLE/PL and TR-PL data from which the energy gap/PL peak and average PL lifetimes are extracted, are displayed in Figures S4A,C, S5A, respectively.

The plots reveal clear trends, namely a moderate but monotonic blue-shift of the energy gap and the PL peak by ~50 meV, a respective increase by a factor of ~2 in the PL QY and a dramatic quenching of the average PL lifetime by an order of magnitude as NC content in the PMMA membranes increases from 0.05 to 5% wt. It can also be observed that the fibers containing a higher NC density exhibit luminescence characteristics that resemble more the respective characteristics of the reference NC films. Insight into the interpretation of the concentration-dependent properties of the fibers is provided by control experiments on PMMA:LHP NC blend films, where the respective emission characteristics are monitored as a function of the polymer:NC mass ratio. The observed trend in PL lifetime and emission QY, displayed in Figure S6, matches well with the respective dependencies observed in Figures 4B,C for the NC-sensitized fibers.

Based on all the experimental evidence, we can interpret the photophysical changes of the NC properties upon electrospinning into the PMMA fibers via a combination of concentration-dependent NC stabilization and dielectric effects. The latter result in emission blue-shift and an increase of the PL quantum yield upon NC insertion into the low dielectric polymer environment, while the former yields a bathochromic shift and dampening of the NC emission at the limit of low NC content. In particular, dielectric screening is nominally expected to increase the exciton binding energy and the oscillator strength relative to pristine films (Takagahara, 1993), blue-shifting the band edge and increasing the radiative recombination rate, thus overall enhancing the emission quantum yield. Such effects are evident in the fibers for NC:polymer weight ratios higher than 1% wt and in the polymer:NC blend films for a concentration higher than 30% wt, as observed in Figure 4 and Figure S6, respectively. On the other hand, as the NC content in PMMA fibers or solid matrixes decreases to lower levels, the monotonic quenching of the QY and the concomitant dramatic increase of the average PL lifetime observed indicate a significant reduction in the FAPbBr_3_ NC radiative recombination rate. Indeed, the exponential fits of the decays indicate that the fast recombination decay channel attributed to radiative recombination reduces substantially in favor of the two other longer decay channels for low NC content, effectively lengthening the average PL lifetime. The quenching of radiative recombination is attributed to a reduced stabilization of the NCs when inserted at low concentrations in the PMMA matrix. At low concentrations, the ligands from the NC surface tend to desorb faster, due to the dilution effect shifting equilibrium toward desorption. This is a common problem for colloidal NCs which appears somewhat pronounced for perovskite NCs due to the more dynamic and looser binding equilibrium (De Roo et al., 2016). It is interesting that the optical manifestation of the NC degradation appears at a significantly lower NC content in the PMMA-NC fibers compared to the respective blend films, which indicates an improved structural and chemical integrity of the perovskite NCs upon polymer fiber encapsulation.

### PVP-LHP NC Fibers

The NC loading of the electrospun PMMA fibers was limited by the use of different solvents employed to dissolve the PMMA and perovskite NC components, namely chloroform and toluene, respectively. For solvent mixtures containing up to 5% wt of the toluene NC to chloroform PMMA solution, the formation of a desired emulsion was achieved. Mixtures with a higher ratio of the toluene solution produced emulsions with viscosity properties not suitable for the electrospinning process. To demonstrate NC-sensitized membranes with higher NC loadings for potential textile-based light emitting applications, a different approach was adopted, in which NCs in toluene were simply immersed or coated into hydrophilic cross-linked membranes of the PVP polymer, produced via electrospinning. Before immersion, the PVP fibers were functionalized via an amphiphilic polymer silane coating, allowing efficient wetting of the hydrophilic PVP fibers with the hydrophobic nanocrystal surface. It is noted that the NC loading in the fibers appears stable over a period of a year and no nanocrystal loss is observed as confirmed by repeated measurements of the membrane weight after immersion and drying.

**Figure 5:**
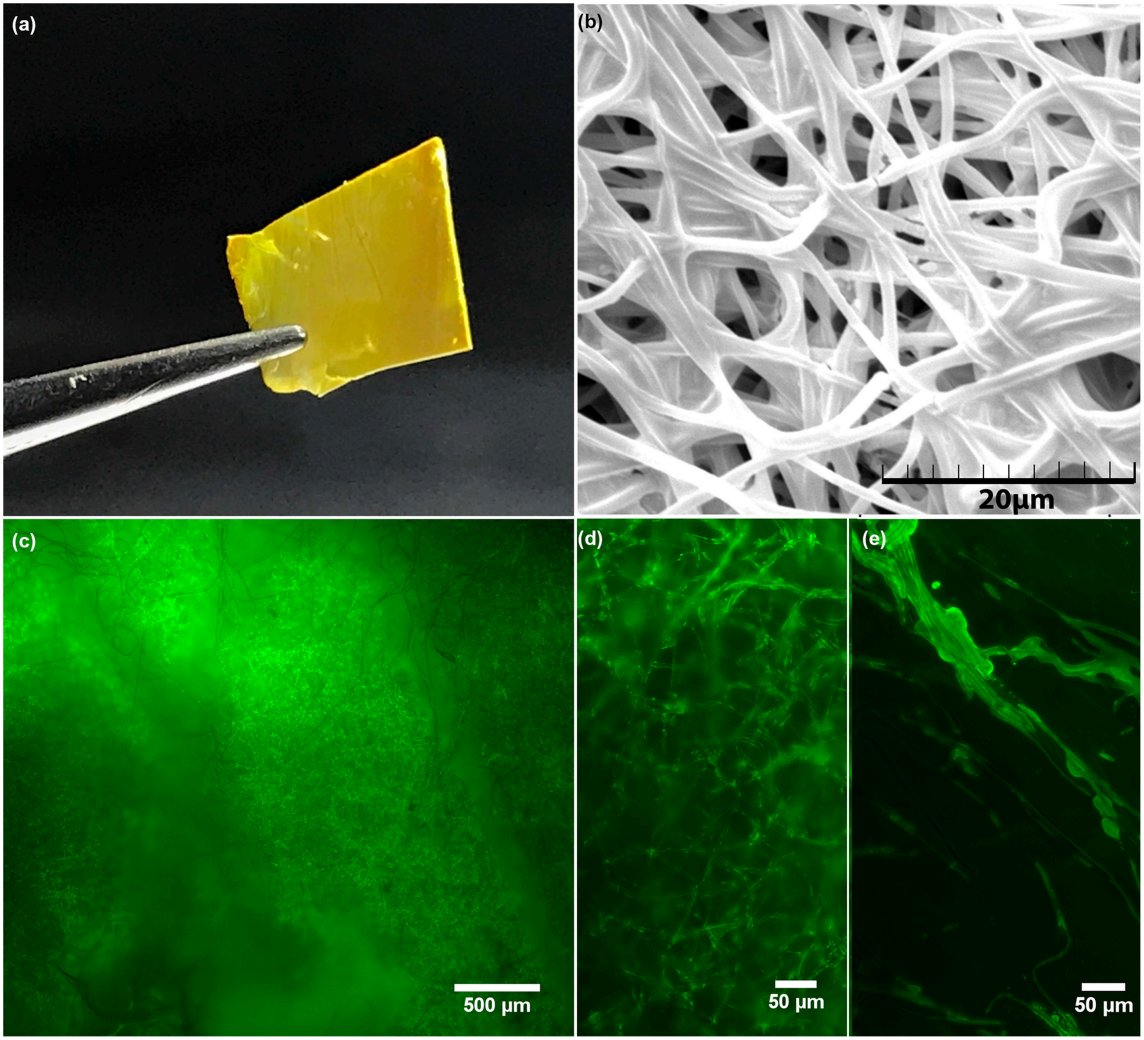
**(a)** Photograph, **(b)** SEM, **(c–e)** Fluorescence microscopy images at different magnifications and different parts; i.e., **(c,d)** at the center, **(e)** at the edge of a FAPbBr_3_ NC-PVP membrane with NC: polymer mass ratio of 50:100 (50%wt).

As seen in Figure 5a, the NC-containing PVP membranes exhibit a paper-like texture and bright yellow-green color as a result of the strong absorption/scattering properties of the incorporated lead bromide perovskite NCs. An SEM analysis yields an average PVP fiber diameter of ~0.70 ± 0.12 μm. Unlike the case of PMMA fibers, both hybrid FAPbBr_3_ NCs and all-inorganic CsPbBr_3_ NCs sensitize uniformly the PVP fibers as seen in Figures 5c,d, with attainable NC loadings for both as high as a 2:1 NC:PVP mass ratio (200% wt); overall, the two PVP-NC fiber types exhibit very similar morphological and optical properties. For high NC content, clustering of the perovskite nanocrystals in between cross-linked fibers and around individual fibers is vividly illustrated in the SEM and fluorescence microscopy images of Figures 5b,e, respectively.

**Figure 6:**
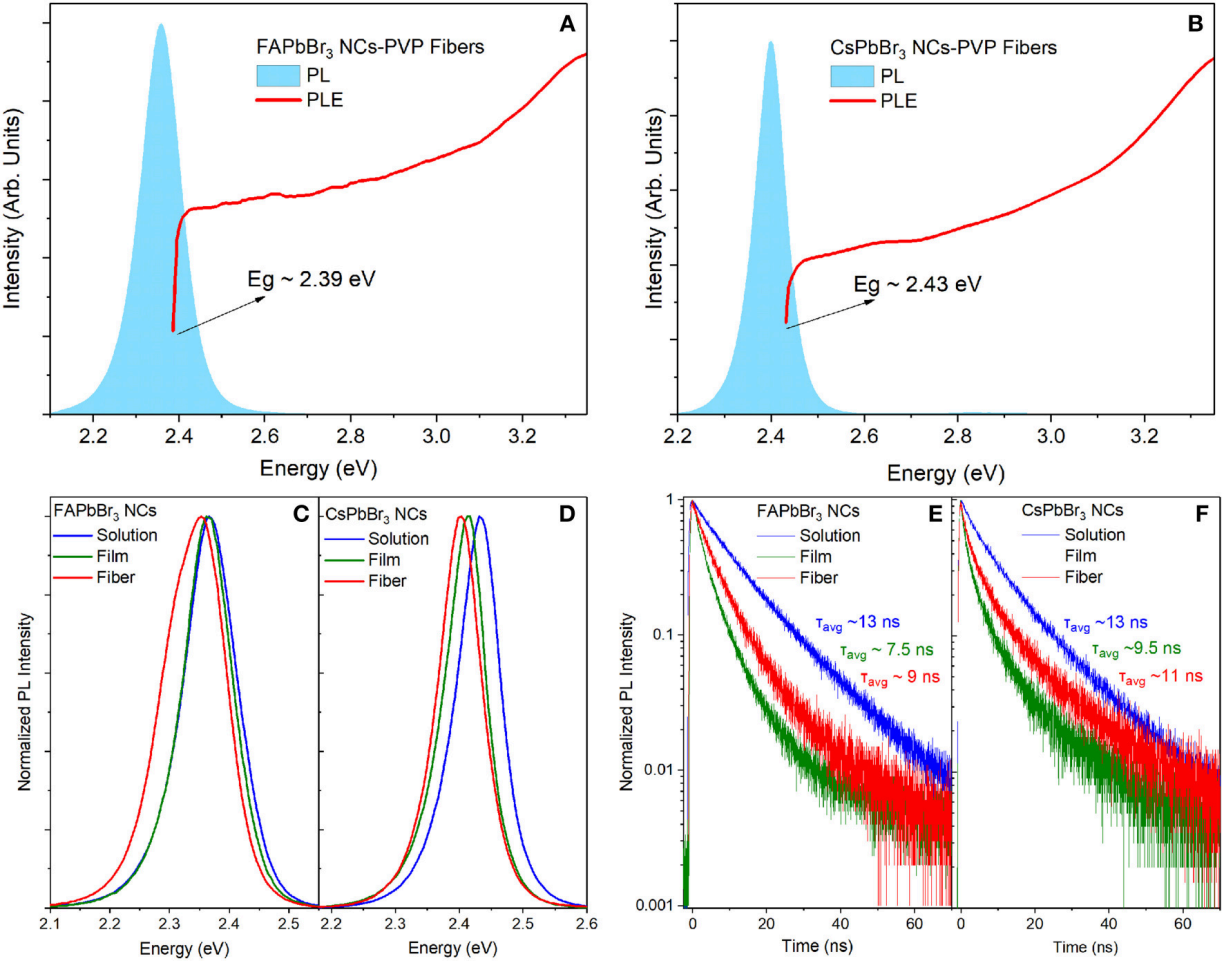
PL and PLE spectra of 30% wt/wt NC:PMMA **(A)** FAPbBr_3_, **(B)** CsPbBr_3_. Continuous wave PL spectra at solution, solid and fibrous state **(C)** FAPbBr_3_, **(D)** CsPbBr_3_. TRPL decays at solution, solid and fibrous state **(E)** FAPbBr_3_, **(F)** CsPbBr_3_.

**Figure 7:**
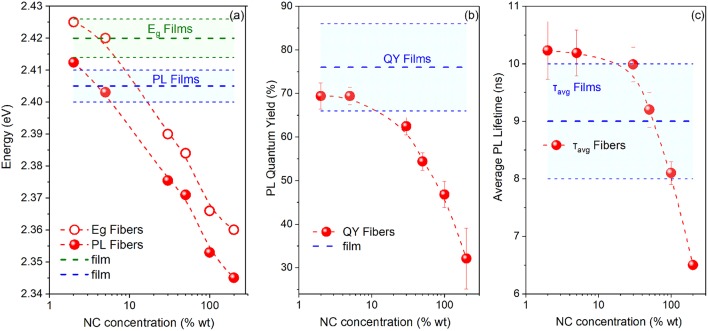
**(a)** Energy gap (E_g_) and PL Peak, **(b)** PL quantum yield and **(c)** PL lifetime vs. NC concentration for the PVP/CsPbBr_3_ NC composite for a wide range of NC content (1–200% wt). Respective characteristics for reference pristine NC films are also displayed.

Representative optical properties of the PVP-NC membranes for the two NC systems presented in Figure 6, Figures S4B,D, S5B, reveal effects similar to those observed in the PMMA-based composites, such as a bathochromically shifted emission and energy gap and triple-exponential PL dynamics with prolonged PL lifetime compared to NC films. Concentration-dependent luminescence properties were probed for the CsPbBr_3_ NC-sensitized PVP fibers over the NC content range of 2–200% and plotted in the summary graphs of Figure 7, with the relevant optical data listed in Table S2.

The optical characteristics of the fibers resemble those of pristine CsPbBr_3_ NC films for NC content up to ~5% wt. For higher NC densities, a monotonic red-shift of the band-edge and quenching of the PL QY and lifetime is observed. QY/Lifetime quenching appears to be promoted via the concentration-dependent shortening of the long decay PL channels and assigned to non-radiative quenching, that assume at low NC at low NC content values of ~8–40 ns while suppressing to values as low as ~3 ns, as the NC concentration increases to the maximum of 200% wt values probed. Based on the aforementioned discussion, we attribute the optical modifications in the PVP type of NC composites, to concentration-dependent aggregation of the NCs. The weak ligand binding into the lead halide perovskite NC surfaces can lead to partial ligand detachment of the ligands from the surface, allowing the NCs to pack and partly merge (Huang et al., 2017; Kovalenko et al., 2017; Akkerman et al., 2018; Zhao et al., 2018), forming larger perovskite nanostructures. It is reasonable to assume that the NC agglomeration effects become increasingly pronounced as NC concentration increases. NC aggregation is consistent with the broadening and red-shift of the band-edge due to the loss of confinement and the decrease of the PL quantum yield and lifetime due to increased non-radiative recombination, as merging of the NCs is known to lead to exciton quenching effects (Stadler et al., 2015; Papagiorgis et al., 2018).

### Air and Water Stability Studies

As mentioned, a prerequisite for the realization of practical perovskite NC optoelectronics is the improvement of the nanocrystal structural, thermodynamic and optical stability under ambient conditions (Huang et al., 2017; Kovalenko et al., 2017; Akkerman et al., 2018; Zhao et al., 2018). Table 1 lists PL QY results for a series of hybrid and inorganic NC fiber and film solids over a yearly period, stored at ambient dark conditions at temperature of ~21°C and humidity levels varied within the 20–40% range through the year. The studied samples were produced out of two parent solutions, one containing CsPbBr_3_ NCs and the other FAPbBr_3_ NCs and their QY was measured at the day of fabrication and after a year under the same experimental conditions. For all samples studied, a moderate red-shift of the PL was observed after a year, while the QY quenching varied widely. In particular, the pristine films of the two NC types exhibit a significant deterioration of their emission efficiency, being more prominent for the CsPbBr_3_ NC system in which emission QY dropped by more than one order of magnitude. In contrast, the QY of the NCs encapsulated with the PMMA fibers was found to quench by <10% of its original value, confirming the suppression of oxygen and moisture transmission to the NCs by the polymer matrix. NCs incorporated within the PVP cross-linked fiber membranes exhibit QY quenching in between the two other solid types, with the PVP-FAPbBr_3_ NC composite exhibiting slightly better stability compared to the PVP-CsPbBr_3_ NC, in agreement with the respective trend observed in the pristine films of the two NC materials. More detailed accelerated aging tests of the materials, at controlled relative humidity and blue-light flux conditions, relevant to industrial requirements for LCD lighting, are to be conducted soon.

**Table 1:** Emission QY stability results for a series of samples in solid and fibrous phase.

**Sample**	**PL QY (%) fresh**	**PL QY (%) 1 year old**
CsPbBr_3_ NCs film	86 ± 8	7 ± 1
FAPbBr_3_ NCs film	83 ± 7	22 ± 4
PVP-CsPbBr_3_ NC fibers (30% wt)	77 ± 6	27 ± 4
PVP-FAPbBr_3_ NC fibers (30% wt)	75 ± 8	36 ± 5
PMMA-FAPbBr_3_ NC fibers (1% wt)	76 ± 5	70 ± 5

**Figure 8:**
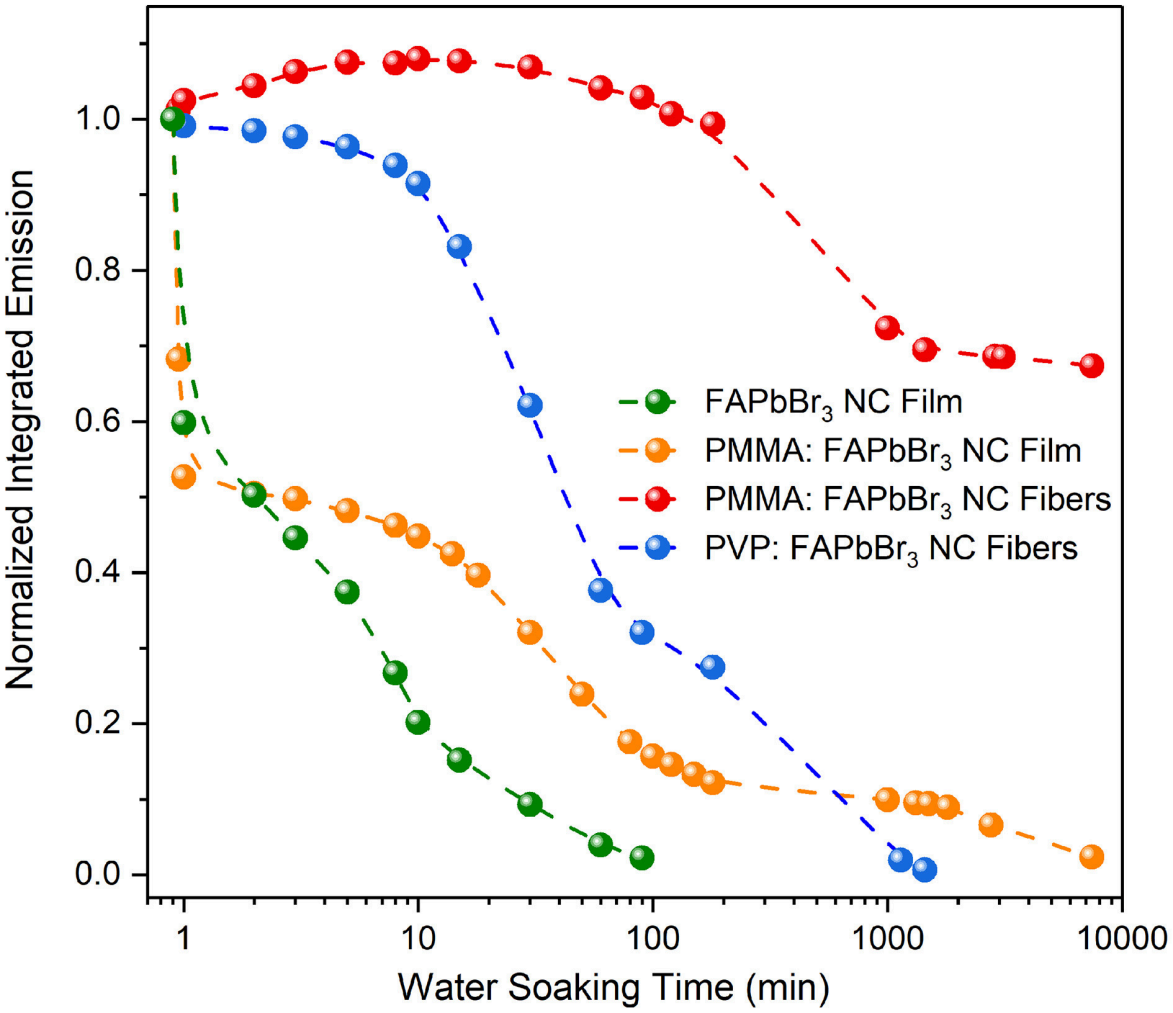
Integrated emission vs. water soaking time for NCs embedded in films and fibers.

Results on the temporal evolution of the NC-integrated luminescence under water soaking conditions for a period of a week (~10^5^ min) are displayed in Figure 8; the raw PL spectra are contained in Figure S7. Four types of samples have been included in the study, namely pristine NC films, PMMA:NC blend films and NC-sensitized PMMA and PVP fiber composites. NCs encapsulated during electrospinning into PMMA fibers exhibit by far the highest optical stability in a water environment, with their integrated emission dropping by <15% after a week of water immersion. Such results compare unfavorably to the highest reported water stability in perovskite NC-polymer fibers, exceeding a period of a month (Wang et al., 2017) but stand on a par (Wang et al., 2016; Tsai et al., 2018) with or even outperform (Liao et al., 2018; Lin et al., 2018) other reported work on the field. Interestingly, a small but reproducible improvement in PL efficiency is observed for the first 5–15 min of immersion for unknown reasons. NCs embedded within the PVP fibers exhibit stable emission over the first ~10–30 min of water immersion with the emission quenching fully after a period of a day. On the other hand, the emission from the pristine NC films is water-suppressed fast, within 100 min of immersion. NCs at low content (1% wt) dispersed within a PMMA film are more efficiently protected by the matrix, with the emission quenching by 80–90% over the first 100 min of soaking, followed though by a much slower longer-term degradation.

## Conclusions

We demonstrate robust polymer-LHP NC fiber composite emitters via (i) encapsulation of the NCs into PMMA hydrophobic fibers using an electrospinning process and (ii) coating hydrophilic cross-linked polyvinylpyrrolidone (PVP) fibrous membranes with NCs via simple immersion and drying processes. For the larger range of the studied content, NCs embedded in the polymer fibers retain much of their narrow and high quantum yield emission, while degradation phenomena and aggregation effects result in the deterioration of the NC luminescence properties at the low/high limits of the concentration range probed for the PMMA-based and PVP-based emitters, respectively. Despite the quite different structural characteristics of the two composite types, we find that the NC sensitizers exhibit similar luminescence characteristics in terms of emission peak, lifetime and quantum yield within the intermediate, overlapping for the two composites NC density regime; furthermore, qualitatively similar concentration-dependent emission characteristics have been obtained in fiber composites employing NCs with oleic acid ligands that have not been subjected to the DDAB-treatment. The aforementioned statements further confirm the validity and generality of the reported studies. The long-term stability under ambient conditions and the short-term material robustness under harsh water-soaking conditions of the polymer-LHP NC fibers were also investigated. The studies confirm the demonstration of robust and bright polymer-fiber emitters with potential applications in backlighting for LCD displays and textile-based light emitting devices.

## Data Availability

All datasets generated for this study are included in the manuscript and the supplementary files.

## Author Contributions

All authors listed have made a substantial, direct and intellectual contribution to the work, and approved it for publication.

### Conflict of Interest Statement

The authors declare that the research was conducted in the absence of any commercial or financial relationships that could be construed as a potential conflict of interest.
