# Supplementary material for: Robust Hydrophobic and Hydrophilic Polymer Fibers Sensitized by Inorganic and Hybrid Lead Halide Perovskite Nanocrystal Emitters
Source: Front Chem. 2019 Feb 26;7:87. doi: 10.3389/fchem.2019.00087 (PMC6399309; doi:10.3389/fchem.2019.00087)
Supplement: Supplementary file 1 [file Data_Sheet_1.pdf]

Supplementary Information

Robust Hydrophobic and Hydrophilic Polymer Fibers Sensitized by Inorganic and Hybrid Lead Halide Perovskite Nanocrystal Emitters

Paris Papagiorgis[1], Andreas Manoli[1], Androniki Alexiou[1], Petroula Karacosta[1], Xenofon Karagiorgis[2], Georgia Papaparaskeva[2], Caterina Bernasconi[3,4], Maryna I. Bodnarchuk[3,4], Maksym V. Kovalenko[3,4], Theodora Krasia-Christoforou[2], Grigorios Itskos[1]*

[1]Department of Physics, Experimental Condensed Matter Physics Laboratory, University of Cyprus, 1678 Nicosia, Cyprus

[2]Department of Mechanical and Manufacturing Engineering, University of Cyprus, 1678 Nicosia, Cyprus

[3]Empa – Swiss Federal Laboratories for Materials Science and Technology, CH-8600, Dübendorf,, Switzerland

[4]Laboratory for Inorganic Chemistry, Department of Chemistry and Applied Biosciences, ETH Zürich, CH-8093 Zürich, Switzerland

Correspondence:

Dr. Grigorios Itskos

itskos@ucy.ac.cy

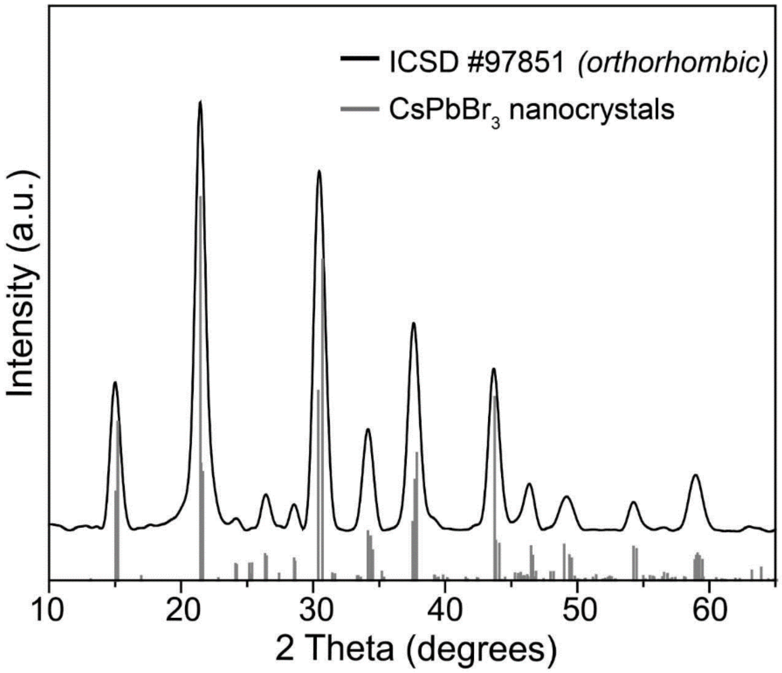

Figure S1: Powder XRD data from the DDAB-capped $CsPbBr_3$ NCs used in our studies. The XRD pattern indicate that the NCs exhibit an orthorhombic crystal structure.

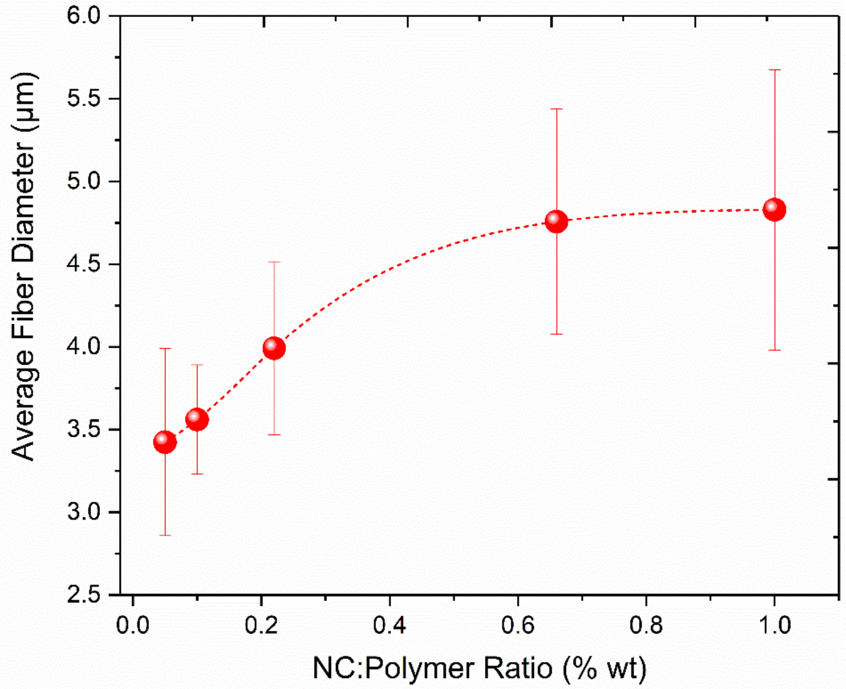

Figure S2: Average fiber diameter versus NC content (% wt) for $FAPbBr_3$/PMMA fiber membranes. The fiber diameters were obtained from software analysis of SEM images.

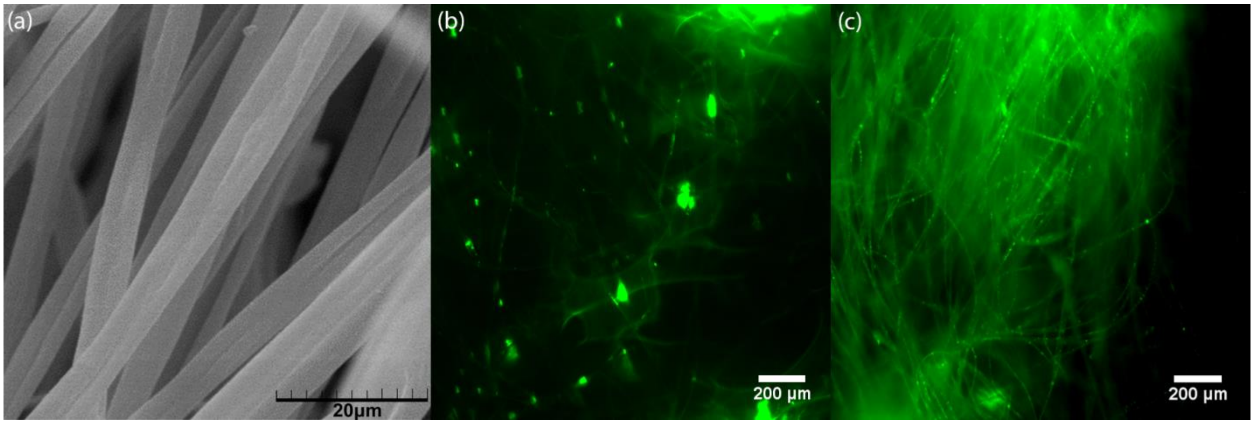

Figure S3: (a) SEM image from a $CsPbBr_3$/PMMA 0.33% wt/wt fiber nanocomposite. Fluorescence microscopy images of **(b)** the aforementioned fibrous membrane. **(c)** a $FAPbBr_3$/PMMA fiber sample with the same NC loading. The images illustrate the higher uniformity in the spatial distribution of the hybrid NCs compared to the Cs-based NCs within the produced PMMA fibers.

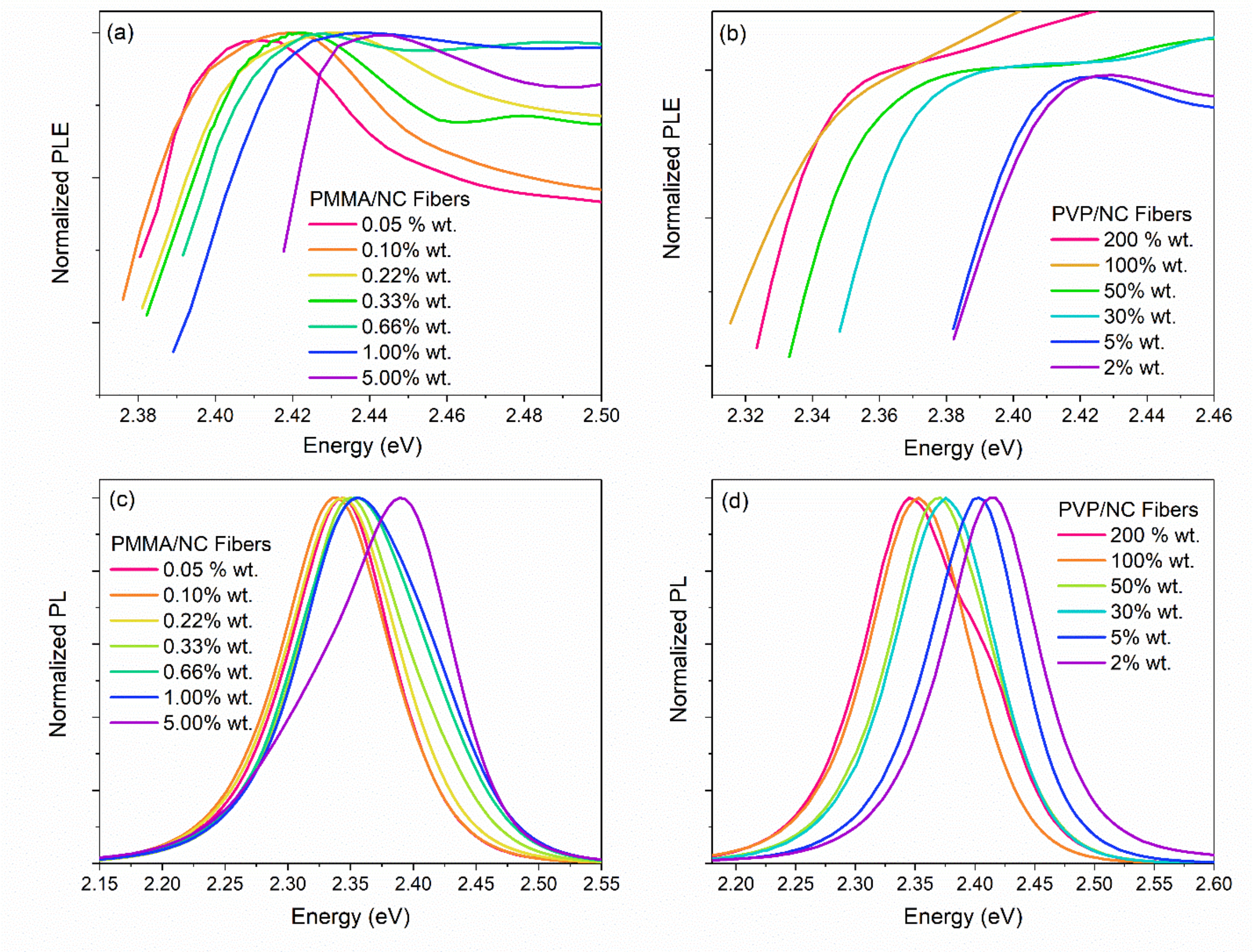

Figure S4: Concentration-dependent PLE experiments from **(a)** PMMA/$FAPbBr_3$ NC and **(b)** PPVP/$CsPbBr_3$ NC fiber composites. The optical gap of the samples was estimated by the respective onset of the PLE signal. PL spectra from the same samples i.e. **(c)** the PMMA/$FAPbBr_3$ NC and **(d)** the PPVP/$CsPbBr_3$ NC fibers.

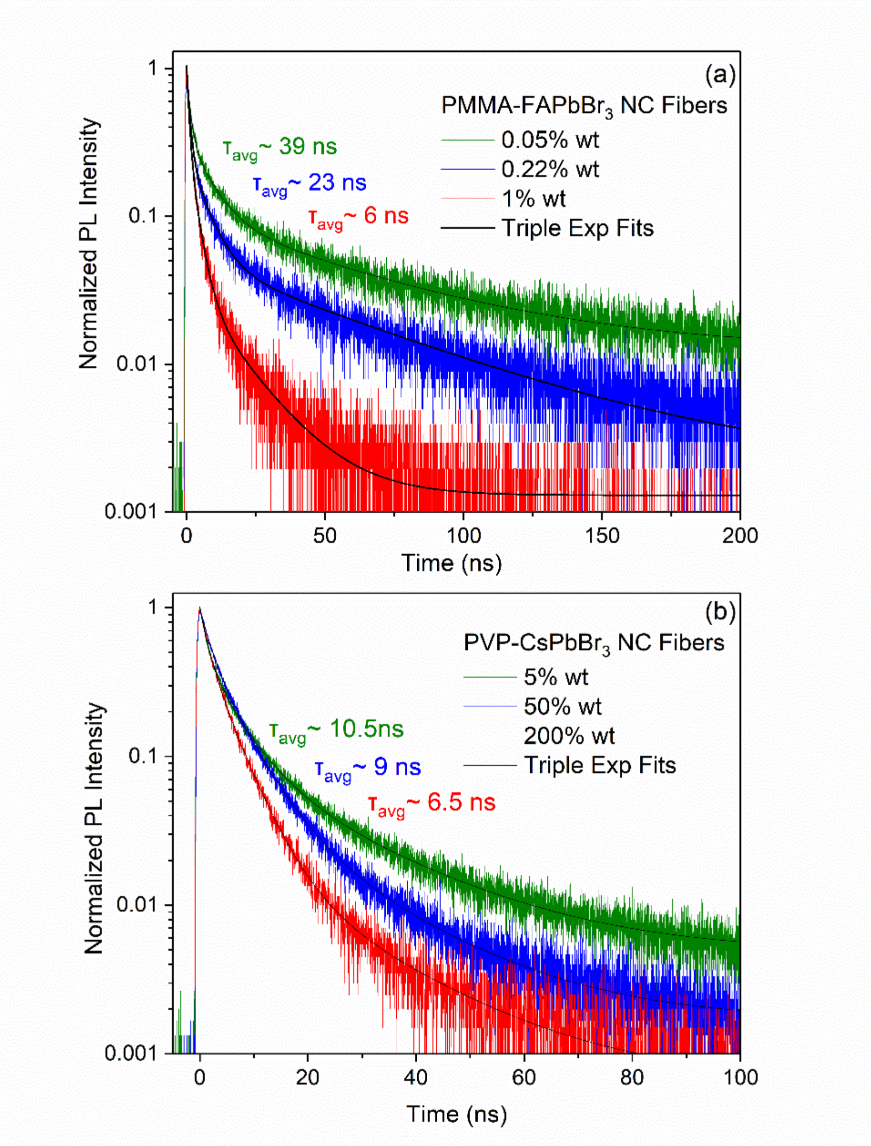

Figure S5: Concentration-dependent PL transients from **(a)** PMMA-$FAPbBr_3$ NC Fibers, **(b)** PVP-$CsPbBr_3$ NC Fibers. Curve fits by a triple exponential model along with the extracted average PL lifetime are also displayed.

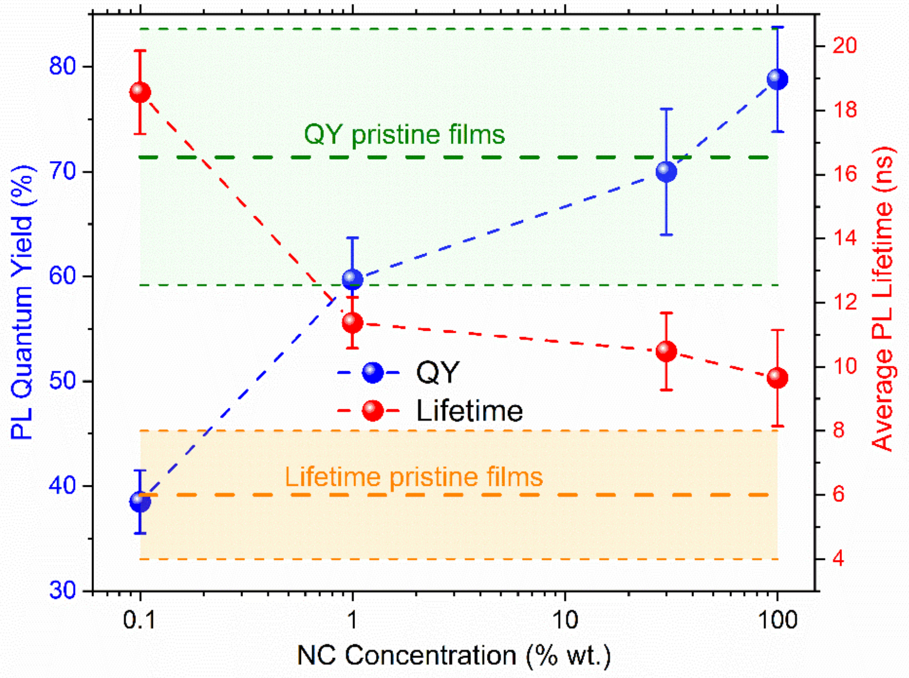

Figure S6: PL quantum yield (blue symbols) and average PL lifetime (red symbols) for various concentrations (% wt) of $FAPbBr_3$ NCs into PMMA matrix films.

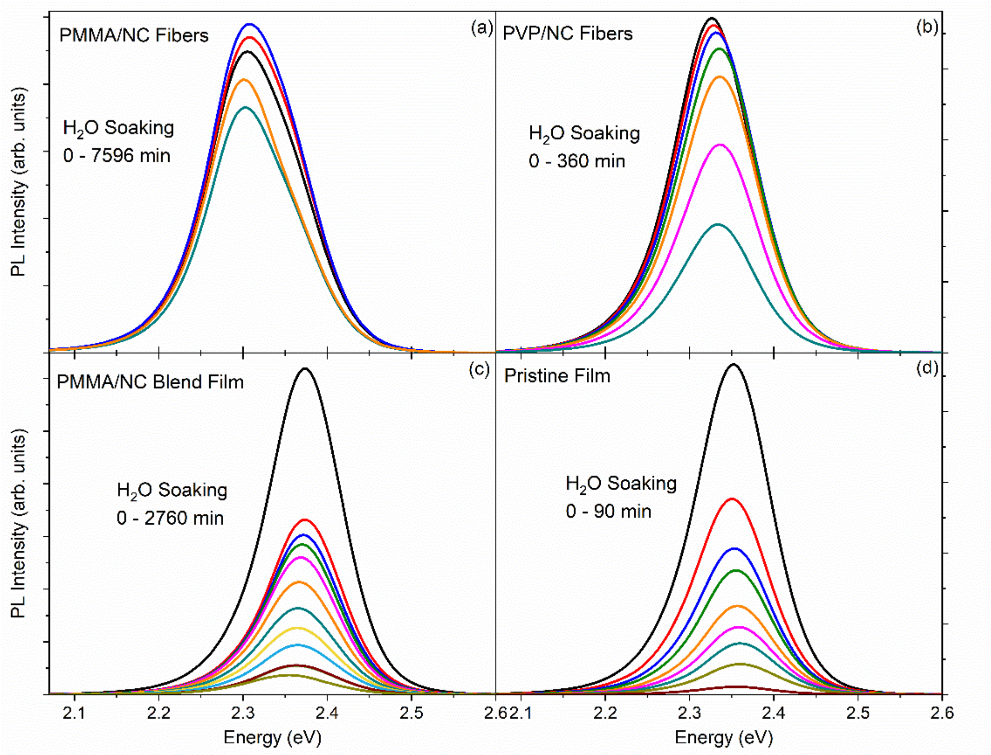

Figure S7: Evolution of the photoluminescence spectra upon water soaking for (a) PMMA/$FAPbBr_3$ NC Fibers, (b) PVP/$CsPbBr_3$ NC Fibers (c) PMMA/ $FAPbBr_3$ NC blend film and (d) pristine $FAPbBr_3$ NC film.

Sample (NC % wt)	Gap (eV)	PL Peak (eV)	PL QY (%)	Stat. Error (%)	PL Av. Time (ns)	Stat. Error (ns)
0.05	2.380	2.339	49	2	39	2
0.1	2.375	2.339	39	3	36	1.6
0.22	2.379	2.337	51	2	23	1.5
0.33	2.383	2.320	60	2	8.0	1.2
0.66	2.387	2.318	70	3	9.0	1.3
1	2.389	2.326	68	6	6.0	1.1
5	2.431	2.389	82	4	6.2	1.4
Film	2.421	2.367	71	12	5.8	1.2

Table S1: Optical data for PMMA/FAPbBr3 NC fibers and films, included in Figure 4 of the manuscript.

Sample (NC % wt)	Gap (eV)	PL Peak (eV)	PL QY (%)	Stat. Error (%)	PL Av. Time (ns)	Stat. Error (ns)
2	2.425	2.412	89	3	10	0.5
5	2.420	2.403	90	2	10	0.4
30	2.390	2.375	77	2	9.8	0.3
50	2.384	2.371	73	2	9.2	0.3
100	2.366	2.353	63	3	8.1	0.2
200	2.360	2.345	37	7	6.5	0.2
Film	2.421	2.405	76	12	9.2	2

Table S2: Optical data for PVP/CsPbBr3 NC membranes and films, included in Figure 7 of the manuscript.
